# Influence of lens opacities and cataract severity on quantitative fundus autofluorescence as a secondary outcome of a randomized clinical trial

**DOI:** 10.1038/s41598-021-92309-6

**Published:** 2021-06-16

**Authors:** Gregor S. Reiter, Luca Schwarzenbacher, Daniel Schartmüller, Veronika Röggla, Christina Leydolt, Rupert Menapace, Ursula Schmidt-Erfurth, Stefan Sacu

**Affiliations:** 1 Christian Doppler Laboratory for Ophthalmic Image Analysis, Department of Ophthalmology and Optometry, Medical University of Vienna, Vienna, Austria; 2 Vienna Clinical Trial Center (VTC), Department of Ophthalmology and Optometry, Medical University of Vienna, Vienna, Austria; 3 Department of Ophthalmology and Optometry, Medical University of Vienna, Währinger Gürtel 18-20, 1090 Vienna, Austria

**Keywords:** Lens diseases, Retinal diseases

## Abstract

The aim of this study is to investigate the impact of age-related lens opacities and advanced cataract, quantified by LOCS III grading, on quantitative autofluorescence (qAF) measurements in patients before and after cataract surgery. Images from a randomized controlled trial evaluating the impact of femtosecond-laser assisted cataract surgery (FLACS) on retinal thickness were analyzed post-hoc. One-hundred and twenty eyes from 60 consecutive patients with age-related cataract were included and assessed with qAF and optical coherence tomography (OCT) before, 1, 3 and 6 weeks after cataract surgery (randomized 1:1 to FLACS or phacoemulsification). LOCS III grading was performed before surgery. Pre- to post-surgical qAF values, as well as the impact of LOCS III gradings, surgery technique, gender, axial length and age on post-surgery qAF values was investigated using generalized linear mixed models. For this analysis, 106 eyes from 53 patients were usable. No difference in qAF was found between FLACS and phacoemulsification (p > 0.05) and results were pooled for the total cohort. Mean pre-surgical qAF was 89.45 ± 44.9 qAF units, with a significant mean increase of 178.4–191.6% after surgery (p < 0.001). No significant difference was found between the three follow-up visits after surgery (p > 0.05). Higher LOCS III cortical opacity quantifications were associated with a significantly greater increase in qAF after surgery (estimate: 98.56, p = 0.006) and nuclear opacities showed a trend toward an increased change (estimate: 48.8, p = 0.095). Considerable interactions were identified between baseline qAF and cortical opacities, nuclear opacities and posterior subcapsular opacities, as well as nuclear opacities and cortical opacities (p = 0.012, p = 0.064 and p = 0.069, respectively). Quantitative autofluorescence signals are significantly reconstituted after cataract surgery and LOCS III gradings are well associated with post-surgical qAF values. Careful consideration of age-related lens opacities is vital for the correct interpretation of qAF, especially in retinal diseases affecting the elderly.

ClinicalTrials.gov Identifier: NCT03465124.

## Introduction

Even in the era of optical coherence tomography (OCT), fundus autofluorescence (FAF) imaging remains an important tool in differentiating and monitoring retinal diseases^1^. The majority of autofluorescent signal, when using blue-light (488 nm excitation) FAF imaging, arises from retinal pigment epithelium (RPE) fluorophores, in particular from lipofuscin and in small amounts (approx. 10%) from melanolipofuscin^2,3^. In normal aging, lipofuscin accumulates until the 7th decade of life^4,5^. The course in more advanced age is not fully explored and the autofluorescence signal increase might slow down or start to decrease with age^4,6^.

Quantification of FAF intensity can be achieved with quantitative autofluorescence (qAF). Therefore, a confocal scanning laser ophthalmoscope (cSLO) is equipped with an internal autofluorescent reference^7^. Gray levels of the image and reference are used to calculate standardized quantifications of the FAF signal with good repeatability in healthy and pathologically aged eyes^8,9^. Using this standardized method, retinal diseases with accumulation of fluorophores can be identified and differentiated from retinal diseases losing fluorophores, i.e. age-related macular degeneration^10–12^.

However, several aspects must be considered when using and interpreting blue-light FAF (488 nm excitation). Macular pigment is blocking the blue-light FAF signal in the fovea and macular diseases can be challenging to interpret. This could be overcome by using green-light FAF (512 nm excitation), which is less affected by macular pigment, but not yet quantifiable^13^. Another aspect is the increasing density of the human lens with age^14^. Although cataract surgery, and thereby removal of the opacified lens and implantation of a clear intraocular lens (IOL), can be performed, age-related macular diseases might emerge earlier than the necessity of surgery, still under the condition of lens opacities. Therefore, attempts have been made to compensate for the lost signal based on lens density and lens opacities^15–17^. The Lens Opacities Classification System (LOCS) III grading is an established, standardized system to grade and compare the severity of cataract^18^.

After the introduction of qAF in 2011, an age-dependent correction factor for qAF outcomes in phakic eyes has been computed to compensate for these age-related lens opacity signal attenuations^15^. Except for severe cataract, the impact of the lens opacities on the qAF intensity therewith was eliminated^19^. However, the precise association between qAF values and individual lens opacity quantifications remains unresolved. This study was conducted to investigate the impact of LOCS III quantifications in subjects with otherwise no ophthalmological conditions, except for age-related cataract on qAF imaging, and to investigate qAF intensities before and after cataract surgery.

## Methods

This study was single center, prospective, randomized, open-label, patient-blinded, parallel-group clinical trial conducted at the Medical University of Vienna from March 2018 to May 2019 assessing the difference in central macular thickness (primary outcome) between femtosecond-laser assisted cataract surgery (FLACS) and phacoemulsification. The Ethics Committee of the Medical University of Vienna approved the study protocol (approved on 01/03/2018) and the study was conducted adhering to the Declaration of Helsinki including current revisions and the Good Clinical Practice (GCP) guidelines. The trial is registered at ClinicalTrials.gov (NCT03465124, registered on 14/03/2018). Patients were included after giving written informed consent. Inclusion criteria comprised any form of age-related cataract in both eyes and an age between 40 and 90 years. In addition, preoperative pupil size > 6.5 mm had to be reached in pharmacological mydriasis. Patients were excluded if any ocular disease except age-related cataract was present or if any previous surgery has been performed. At each visit a best-corrected visual acuity (BCVA) examination was performed before pupil dilation with eye drops containing 0.5% tropicamide and 2.5% phenylephrine. Cataracts were graded after pupil dilation to at least 7 mm and spectral domain optical coherence tomography (SD-OCT) and quantitative autofluorescence (qAF) was assessed as a secondary outcome (both Spectralis HRA + OCT, Heidelberg Engineering, Heidelberg, Germany). Each patient was scheduled before surgery and 1, 3 and 6 weeks after surgery. Both eyes of the same patient underwent cataract surgery on the same day and the same surgeon performed all surgeries for all eyes. Eyes were 1:1 randomized beforehand to either phacoemulsification or femtosecond-laser assisted cataract surgery (FLACS, FEMTO LDV Z8, Ziemer Ophthalmic Systems, Port, Switzerland) and each patient received phacoemulsification in one eye and FLACS in the other eye. Randomizations were performed using a computer-generated list with beforehand allocated numbers, generated with Datainf Randlist software A (version 2.0, Datainf GmbH) by an investigator (L.S.) and stored individually in a non-transparent envelope. The envelopes were stored by an independent researcher until they were first opened in the operating room by the surgeon (R.M.). The patient remained blinded for the allocation of treatment arms. In all eyes the same clear intraocular lens without blue-light filter (Vivinex XC1, Hoya Corporation, Tokyo, Japan) was inserted. Post-surgery treatment was prescribed as Ketorolac eye drops three times a day for 3 weeks and Dexamethasone with Gentamicin three times a day for 7 days. No changes to the trial design or trial outcomes were made after the start of the trial.

### Sample size calculation

Sample size was calculated for the primary outcome of this clinical trial—central macular thickness: a clinical relevant change of macular thickness was defined as 2 µm with a standard deviation of 5.2 based on the results by Gharbiya et al. (change of macular thickness after 1 and 2 month, respectively)^20^. For a paired *t* test, given a power of 80% and a significance level of 0.05 with possible dropouts, 60 eyes per group (= 60 pairs or 120 eyes in total) are needed for the primary outcome.

### Cataract grading and pre-surgical examination

All eyes were graded for age-related cataract by an experienced grader (L.S.) using the Lens Opacities Classification System (LOCS) III^18^. All examinations were performed using the Haag-Streit 900 Slit Lamp biomicroscope (Haag-Streit AG, Koeniz, Switzerland). Nuclear opalescence (NO) and nuclear color (NC) were graded ranging from 0.1 to 6.9. Cortical opacities (C) and posterior subcapsular opacities (P) were graded ranging from 0.1 to 5.9. Ocular axial length was measured using the IOLMaster 500 (Carl Zeiss Meditec AG, Jena, Germany).

### Retinal imaging

Twenty by twenty degrees SD-OCT scans were acquired on all visits. Each scan was centered on the fovea and was acquired with 1024 A-scans and 49 B-scans. Quantitative autofluorescence imaging was performed using the available system using a confocal scanning laser ophthalmoscopy (cSLO) unit and a light excitation wavelength of 488 nm. The device was equipped with an internal fluorescence reference, provided and calibrated by the manufacturer and a light transmission filter tolerating light wavelengths between 500 and 680 nm. A detailed description of this method has been published^7,21^. Room lights were switched off for the examination and the infra-red mode was used to align the camera on the fundus. The patients were warned about the blue light and the qAF mode was subsequently activated. The camera was aligned to reach maximum signal and sharpness throughout the fundus without attenuation at the corners. A bleaching period of at least 20 s using the 488 nm light was performed to minimize light absorption by rhodopsin. The patient was then asked to blink once and a qAF series of 12 frames (30° × 30°, 768 × 768 pixels) was subsequently acquired. A second series of the same eye was acquired after a short break of one minute. The series was immediately reviewed after acquisition for quality and artifacts (i.e. eye lids and lashes) and if insufficient (a minimum of 9 useable frames had to be available per series), qAF imaging was re-performed. A detailed report on how to acquire qAF images has been published and is available for further information^21^.

### Image assessment

**Figure 1:**
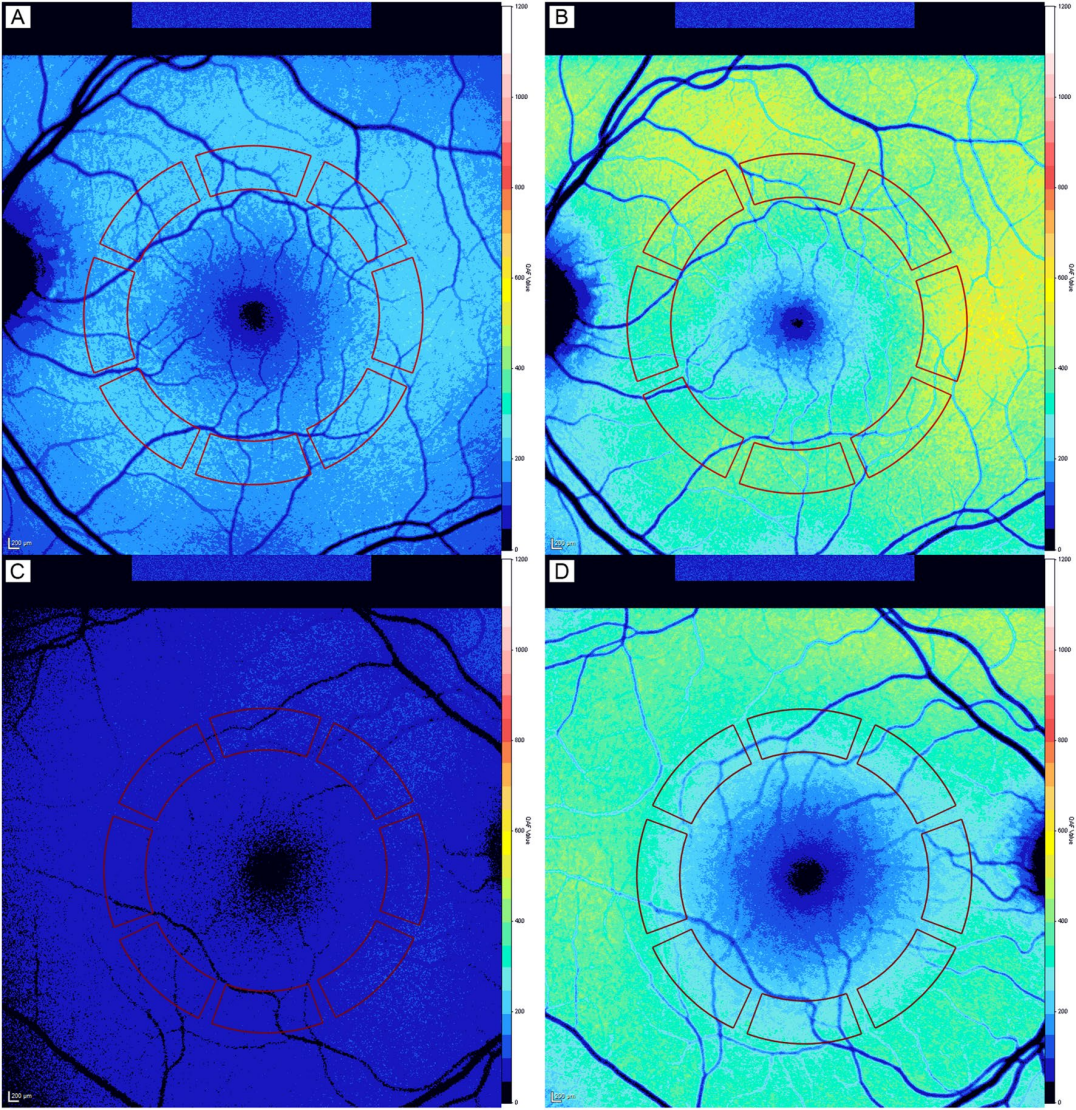
Quantitative autofluorescence (qAF) imaging of a 64.9 years old, male participant before (**A** + **C**) and 6 weeks after (**B** + **D**) cataract surgery. qAF_8_ was quantified as the mean of the middle 8-segment ring of the Delori-pattern (red pattern). The top row shows the left eye with a LOCS III grading of (NO = 2/NC = 2/C = 1/P = 1) and the bottom row shows the right eye of the same patient with a LOCS III grading of (NO = 3/NC = 3/C = 2/P = 3). Images were acquired and analyzed with the Heidelberg Eye Explorer (HEYEX) Version 1.9.10.0 (Heidelberg Engineering, Heidelberg, Germany).

All qAF series were analyzed using the HEYEX software, provided by the manufacturer (Version 1.9.10.0, Heidelberg Engineering, Heidelberg, Germany). All frames of the series were checked and up to 3 frames were removed if quality was insufficient or artifacts were visible. At least 9 frames had to be available for creating a mean qAF image. A Delori pattern was then centered on the fovea, which was aligned with the support of the SD-OCT scan. The Delori-pattern consists of the fovea and a 4-segment ring followed by three 8-segment rings. The mean qAF of the middle 8-segment ring of the Delori-pattern (qAF_8_) was further used for quantitative analysis (Fig. 1). This ring’s mean radius is approximately at 8.4° visual angle with a thickness of 2.5°^22^. Signal blocking pixels by retinal vessels were automatically detected and excluded by the software. No manual correction of the automatic threshold for vessel detection was executed. No correction of qAF due to the opacified lens was performed before surgery to assess the impact of age and lens opacifications and all eyes were labeled as pseudophakic after surgery.

### Statistics

All data are stated as mean ± standard deviation (SD), unless stated otherwise. IBM SPSS Statistics 26 (IBM Corp., Armonk, New York, USA) was used to execute statistical testing. A generalized linear mixed model (GLMM) was calculated to investigate a difference between the qAF intensity before surgery and 1, 3 and 6 weeks after surgery. Because both eyes were eligible for study inclusion, the random effect for the factor ‘eye laterality’ was nested in the patients’ ID. The difference between the effects of the randomized surgical method (phacoemulsification vs. FLACS) on the qAF was investigated as a fixed effect in this model. A second GLMM was built to investigate the importance and effect of pre-surgical parameters on the change of qAF. The change of qAF between the pre-surgical assessment and the 6-week post-surgical assessment was defined as the independent variable. The latest available assessment was used to minimize influences from the surgical and the post-surgical treatment procedure. Again, the random effect for the factor ‘eye laterality’ was nested in the patients’ ID. All other parameters (sex, age, surgical technique, axial length, NO, NC, N and P from the LOCSIII grading, qAF baseline value and the interactions between the LOCSIII lens parameters and the interactions between the LOCSIII lens parameters and the qAF baseline value) were investigated as fixed effects in the model. The best model was identified using the corrected Akaike information criterion (AICc). The AICc assesses the goodness of fit of the statistical model but also takes over-complexity into account. It only serves as a comparison between models^23^. Therefore, a GLMM with the random effects only was calculated and the GLMM including the fixed effects was adapted and re-calculated omitting the least significant parameter until the difference in AICc between the two models was smaller than 2. The significance level α was set to 0.05. No correction for multiple testing was performed due to the exploratory cause of this study.

## Results

**Figure 2:**
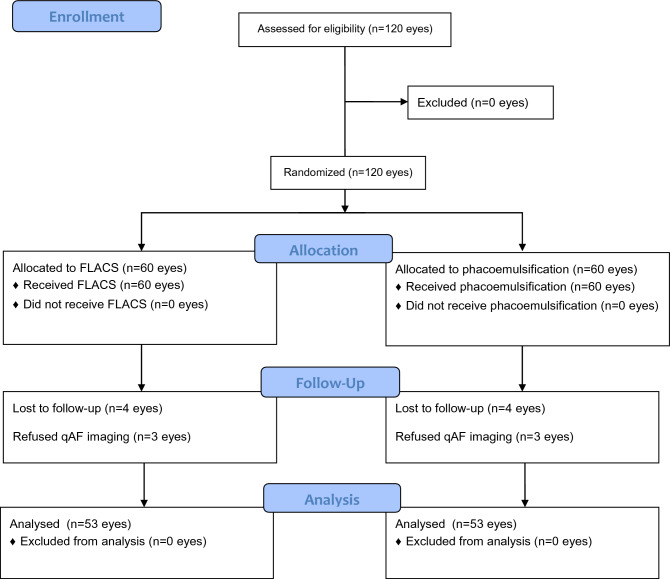
Flow diagram of patients randomized into either femtosecond-laser assisted cataract surgery (FLACS) or phacoemulsification. Because each patient received FLACS in one and phacoemulsification in the other eye, the numbers of eyes do not differ between the two treatment arms.

One hundred twenty eyes from 60 patients were prospectively enrolled. The participant flow is shown in Fig. 2. Four patients (4 eyes per group, in total 8 eyes) were lost to follow-up after surgery and were therefore excluded from analysis. Three patients (3 eyes per group, in total 6 eyes) refused qAF assessment at baseline and all follow-up visits. One hundred six eyes from 53 patients completed the study including qAF assessments. The mean age at baseline was 70.9 ± 7 years (range 53.5–84.9 years). Thirty-five patients (66%) were female. The pooled mean ocular axial length was 23.73 ± 1.26 mm. The pooled mean values for the LOCSIII lens parameters, NO, NC, C and P were 2.5 ± 1.2, 2.5 ± 1.2, 2.2 ± 1.0 and 1.2 ± 1.5, respectively. The summarized demographic data between the randomized arms are shown in Table 1.

**Table 1:** Demographic data of eyes randomized into each treatment arm and for the total cohort. Data are means ± standard deviations or numbers (%). *FLACS* femtosecond-laser assisted cataract surgery, *LOCS* Lens Opacities Classification System, *qAF* quantitative autofluorescence.

	FLACS (n = 53 eyes)	Phacoemulsification (n = 53 eyes)	Total cohort (n = 106 eyes)
Age (years)	70.9 ± 7	70.9 ± 7	70.9 ± 7
Sex (Female)	35 (66%)	35 (66%)	35 (66%)
Laterality (Left eye)	27 (51%)	26 (49%)	53 (50%)
Axial length (mm)	23.77 ± 1.33	23.73 ± 1.3	23.73 ± 1.26
Baseline qAF (qAF units)	86.49 ± 44.09	92.41 ± 45.92	89.45 ± 44.9
**LOCS III gradings**			
Nuclear color (NC)	2.51 ± 1.23	2.47 ± 1.20	2.5 ± 1.2
Nuclear opalescence (NO)	2.51 ± 1.23	2.45 ± 1.22	2.5 ± 1.2
Cortical (C)	2.21 ± 1.0	2.09 ± 0.99	2.2 ± 1.0
Posterior subcapsular (P)	1.32 ± 1.58	1.06 ± 1.38	1.2 ± 1.5

### Post-surgical qAF_8_ progression

**Figure 3:**
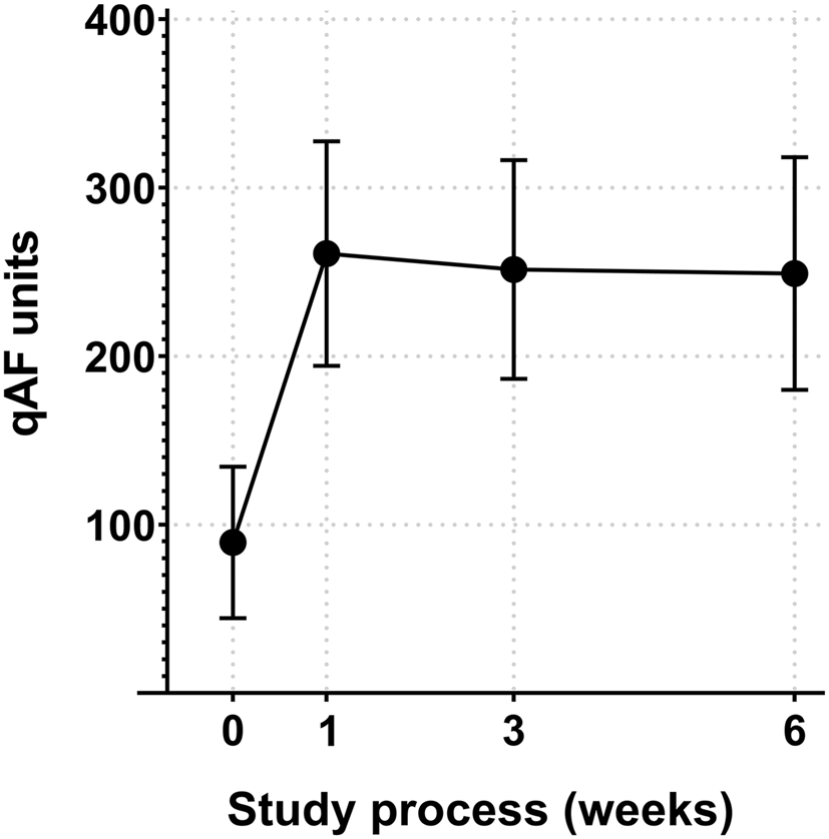
Course of quantitative autofluorescence (qAF) before and after cataract surgery demonstrated by the mean and 95% confidence intervals. qAF assessment was performed before cataract surgery (week 0) and at week 1, 3 and 6 after surgery.

No significant difference in qAF was found between the surgical procedures (phacoemulsification vs. FLACS, p = 0.22), therefore all values were pooled for further calculations regarding qAF. The mean initial, pre-surgical qAF_8_ was 89.45 ± 44.9 qAF units. Mean qAF_8_ for 1, 3 and 6 weeks after surgery was 260.85 ± 66.7 qAF units, 251.48 ± 64.9 qAF units and 249.02 ± 69.04 qAF units, respectively. This results in a mean gain of qAF units of + 191.6%, + 181.1% and + 178.4% for 1, 3 and 6 weeks after surgery, respectively. A statistically significant difference in qAF_8_ was found between the baseline visit and all three consecutive visits (p < 0.001). No significant difference in qAF_8_ was found between each of the follow-up visits (week 1 to 6, all p > 0.05). The course of qAF during the study is shown in Fig. 3.

### Importance and effects of baseline parameters

The calculation of the GLMM was started using all random and fixed effects as described in the method sections. Because the interaction between NO and NC was high, both of these factors could function as a substitute of the other. Therefore, NC was removed from the model and NO was used as the parameter for the severity of the nuclear involvement. The full model reached an AICc of 925.2. No further improvement (> 2) of the model was reached after trying to omit the least significant parameter (P*qAF baseline value (Interaction), p = 0.862; AICc after omitting: 923.61). A second GLMM was calculated only using the random effects (eye laterality nested in patient ID). The AICc reached a value of 1033.64. A difference of AICc of 108.42 between the full model and the model only including random effects was reached. A full listing of all parameters in the model and their estimates is shown in Table 2. The most significant factor was specified by the positive association between cortical opacities and change of qAF (p = 0.006, Table 2). This indicates a higher postoperative change in qAF with higher cortical opacities grading.

**Table 2:** Estimates of the fixed effects of the best fitted mixed model with a corrected Akaike information criterion (AICc) of 925.2. Estimates are given for the change in qAF units before and after cataract surgery. *CI* confidence interval, *FLACS* femtosecond-laser assisted cataract surgery, *NO* nuclear opacities, *C* cortical opacities, *P* posterior subcapsular opacities, *qAF* quantitative autofluorescence.

Parameter	Estimate	Significance	95% CI
Constant	196.35	p = 0.322	− 195.75 to 588.45
Gender (male)	− 13.72	p = 0.461	− 50.7 to 23.19
Surgical technique (FLACS)	− 15.86	p = 0.254	− 43.37 to 11.65
Ocular axial length	− 8.64	p = 0.243	− 23.27 to 6
NO	48.8	p = 0.095	− 8.77 to 106.37
C	98.56	p = 0.006	29.4 to 167.71
P	20.69	p = 0.417	− 29.77 to 71.15
Age	0.91	p = 0.408	− 1.27 to 3.08
qAF baseline value	− 0.14	p = 0.830	− 1.45 to 1.17
NO*C (interaction)	− 15.68	p = 0.069	− 32.6 to 1.25
NO*P (interaction)	− 11.76	p = 0.064	− 24.21 to 0.7
NO*qAF baseline value (interaction)	− 0.05	p = 0.795	− 0.4 to 0.31
C*P (interaction)	− 1.81	p = 0.746	− 12.92 to 9.3
C*qAF baseline value (interaction)	− 0.52	p = 0.012	− 0.925 to − 0.12
P*qAF baseline value (interaction)	0.03	p = 0.862	− 0.32 to 0.38

### Safety issues

In both treatment arms, no adverse events were recorded at any time point of this study.

## Discussion

Quantitative autofluorescence is a powerful tool in the diagnostic toolbox of the retinal specialist. However, lens opacities can significantly attenuate the retinal autofluorescent signal. In this study we found that qAF intensities were significantly reconstituted after cataract surgery, in comparison to pre-surgical values (p < 0.001). Post-surgical pseudophakic qAF remained steady in the investigated follow-up period of 6 weeks. The quantitative LOCS III grading of cortical lens opacities was significantly associated with an increased change in post-operative qAF, indicating higher post-operative qAF values with higher pre-surgical cortical opacities grading. A similar estimate was found for nuclear opacities, however, the significance level was not reached (p = 0.095). A further significant factor in the GLMM was the interaction between qAF baseline value and cortical opacities (p = 0.012) with a small negative estimate. Additional trends for two interactions (nuclear and posterior subcapsular opacities, as well as nuclear and cortical opacities) were identified (p = 0.064 and p = 0.069, respectively).

Lens opacities in phakic eyes can significantly reduce blue-light fundus autofluorescent signal using the 488 nm wavelength^24^. Therefore, an age-related correction formula was integrated in the qAF analysis software (HEYEX, Heidelberg Engineering, Heidelberg, Germany)^15^. Previous research in qAF imaging mainly focused on phakic patient without significant lens opacities, even when investigating age-related diseases, i.e. age-related macular degeneration (AMD)^25^. Other research only included pseudophakic eyes for the investigation of AMD^26^, which might exclude younger patients and distort results into a more advanced direction of disease stage. Our group previously included pseudophakic eyes and phakic eyes based on a LOCS III threshold and did not find an impact of lens status (phakic/pseudophakic) after applying an age-related correction in AMD^12,19^. Nonetheless, in these previously published investigations our group had to exclude patients with higher LOCS III grading and qAF signal attenuating lens opacities. As a result, the impact of the LOCS III grading, also for more advanced cataracts, needed further evaluation. Previous investigations for the attenuation of macular pigment assessments due to lens opacities revealed only a weak predictive ability of LOCS III gradings and post-surgical macular pigment measurements^17^. For cataract type, nuclear cataract in particular, but also subcapsular posterior opacities were associated with a higher signal attenuation of macular pigment measurements^24,27,28^. Our results identified cortical cataract to be associated with the gain of qAF signal after surgery. Nuclear cataract on the other hand only showed a trend towards an association with qAF gain (p = 0.095). Cortical cataract might lead to an increased scattering of light. The amount of light scattering, previously indicated by the width of the signal intensity histogram in macular pigment measurements (narrow histogram indicating increased light scattering)^17^, is in agreement with our finding of qAF signal attenuation associated with higher cortical LOCS III gradings. Evaluation of histogram appearances might also be reasonable for qAF measurements.

Mean qAF signal was strongly increased after cataract surgery (178.4–191.6%), highlighting the importance of appropriate correction in patients with lens opacities when quantifying blue-light fundus autofluorescence. On the other hand, no association with qAF changes were found for sex, gender, axial length or age (all p > 0.05). qAF signal was previously described to increase with age^22^. Our research however, investigated the change of qAF after cataract surgery and therefore does not conclude on an age dependence of qAF. The same applies to the other non-significant parameters. The model used in this investigation uses a large amount of parameters. Since an interpretation of such a formula would not be practical, no specific formula has been developed. This investigation has only been conducted to assess qAF changes after cataract surgery. The implementation of a new correction formula needs to be validated for accuracy and practical issues in a second validation study. The previous use of age only for a correction equation of qAF values^15,22^ follows the same polynomial course of increased lens densities with age^4^. LOCS III gradings were significantly associated with qAF change in our study, therefore we recommend considering lens opacities and age before qAF measurements.

After surgery, qAF intensities remained stable without significant differences between the follow-up visit at week 1, 3 and 6 after surgery (all p > 0.05). This is however to be expected and proves good repeatability of qAF imaging in a short-term follow-up, which has been previously described for healthy eyes and eyes affected by retinal diseases^7,9^. Nonetheless, the considerable change of qAF after cataract surgery and clear IOL implantation indicates a serious attenuation of the autofluorescent signal with clinically relevant cataract, which has to be considered in studies investigating retinal diseases in the elderly. To exclude eyes with minor lens opacities seem unreasonable and age-related correction for lens opacities should be performed. The inclusion of only non-opacified eyes or already pseudophakic eyes poses risk of selection bias and the underrepresenting of mid-stage age-related diseases (i.e. intermediate age-related macular degeneration). However, the exclusion of more progressed cataracts is inevitable for precise and high-quality studies including qAF imaging. In a previous study we excluded patients with the LOCS III grading greater than NO = 3, NC = 3, C = 2 and P = 2 and did not find an impact of lens status on qAF measurements after applying the age-related correction equation^19^. However, this cutoff was defined artificially. In regard to our findings, non-tolerance of advanced cortical opacities seems justified.

Therefore, individual examination needs to be performed with extraordinary diligence and LOCS III gradings might be a possibility to decide on specific thresholds. Novel techniques of cataract quantifications based on swept-source OCT technology or Scheimpflug principle might be useful and their impact on qAF have to be evaluated in future studies^29,30^. The LOCS III grading is subjective, but a reliable and easily accessible tool for the evaluation of lens opacities in good agreement to technical-assisted quantifications^30^.

This study has some limitations; The study was powered for its main outcome (differences in macular thickness between FLACS and phacoemulsification). Therefore, parameters showing a trend (i.e. nuclear opacities, p = 0.095) might still reach significance in a larger sample size. In addition, mean subcapsular posterior cataract grading was 1.2, which probably underrepresents the impact of subcapsular posterior opacities in this patient cohort. This study did not account for lens autofluorescence, which has been reported previously to increase with age^31^. The error due to lens autofluorescence is expected to be of minor degree, since age was included in our statistical model. Still, not all of the variance of lens autofluorescence can be accounted by age or the LOCS III grading and this has to be mentioned as a limitation. LOCS III grading has been performed by one experienced grader to ensure consistency throughout the study.

The strengths of this study are its prospective manner with a large sample size compared to previous reported signal attenuations for macular pigment assessments and appropriate consideration of two eyes from the same patient in the statistical model. In addition, only one experienced surgeon performed all surgeries and the same clear IOL was inserted in all eyes. The results are generalizable for a cohort of patients with age-related lens opacities and healthy retinal conditions, but without other media opacities (i.e. cornea). It is however assumed, that these findings are also true for measurements in patients with a pathologically aged retina, because the effects after surgery remained stable over the follow-up period and might therefore be independent from the retinal condition.

In summary, qAF signal was significantly reconstituted after cataract surgery with a mean gain of qAF signal of 178.4–191.6%. Appropriate correction of age-related lens opacities is important for the valid interpretations of qAF. Patients with severe cataract have to be evaluated cautiously and, in case of doubt, preferentially excluded from analysis due to the lack of strong associations with LOCS III gradings. qAF is a powerful tool in the diagnostic toolbox of the retinal specialist. However, correct use and correct interpretation of the results are vital to gain proper insights into retinal diseases.
